# Nutrition vs association: plant defenses are altered by arbuscular mycorrhizal fungi association not by nutritional provisioning alone

**DOI:** 10.1186/s12870-022-03795-3

**Published:** 2022-08-16

**Authors:** Chase A. Stratton, Swayamjit Ray, Brosi A. Bradley, Jason P. Kaye, Jared G. Ali, Ebony G. Murrell

**Affiliations:** 1 The Land Institute, 2440 E Water Well Rd, Salina, KS 67401 USA; 2 Department of Entomology, Pennsylvania State University, University Park, PA 16802 USA; 3 Department of Ecosystem Science and Management, Pennsylvania State University, University Park, PA 16802 USA

**Keywords:** Arbuscular mycorrhizal fungi, Spodoptera frugiperda, Zea mays, Gene expression, Induced defense, Nutrient digests

## Abstract

**Background:**

While it is known that arbuscular mycorrhizal fungi (AMF) can improve nutrient acquisition and herbivore resistance in crops, the mechanisms by which AMF influence plant defense remain unknown. Plants respond to herbivory with a cascade of gene expression and phytochemical biosynthesis. Given that the production of defensive phytochemicals requires nutrients, a commonly invoked hypothesis is that the improvement to plant defense when grown with AMF is simply due to an increased availability of nutrients. An alternative hypothesis is that the AMF effect on herbivory is due to changes in plant defense gene expression that are not simply due to nutrient availability. In this study, we tested whether changes in plant defenses are regulated by nutritional provisioning alone or the response of plant to AMF associations. Maize plants grown with or without AMF and with one of three fertilizer treatments (standard, 2 × nitrogen, or 2 × phosphorous) were infested with fall armyworm (*Spodoptera frugiperda*; FAW) for 72 h. We measured general plant characteristics (e.g. height, number of leaves), relative gene expression (rtPCR) of three defensive genes (*lox3*, *mpi*, and *pr5*), total plant N and P nutrient content, and change in FAW mass per plant.

**Results:**

We found that AMF drove the defense response of maize by increasing the expression of *mpi* and *pr5*. Furthermore, while AMF increased the total phosphorous content of maize it had no impact on maize nitrogen. Fertilization alone did not alter upregulation of any of the 3 induced defense genes tested, suggesting the mechanism through which AMF upregulate defenses is not solely via increased N or P plant nutrition.

**Conclusion:**

This work supports that maize defense may be optimized by AMF associations alone, reducing the need for artificial inputs when managing FAW.

## Background

The majority of land plants form symbiotic relationships with arbuscular mycorrhizal fungi [1–4]. These associations benefit plants in a myriad of biological and ecological gains [5–8]. The most often studied benefit is increased nutrient uptake in AMF colonized plants. However, more recent research has shown that AMF can also alter defense capacity against pathogens and insects [9–13]. For example, colonization of ragwort by AMF coincides with an increase in plant-derived defense compounds and compounds that are not produced otherwise [11]. More generally, AMF are thought to benefit plant defense by priming the plants so defense compounds are present before attacks occur [14, 15].

While some of the benefits can be observed at an organismal scale, the mechanisms by which AMF provide these advantages remain unclear. Indirectly, AMF may alter plant defenses by providing improved nutrition to their host plants [16–19]. Multiple studies have shown that AMF increase uptake of multiple nutrients, including nitrogen and phosphorous [1, 4, 10, 20–22]. Fungal hyphae expand the contact area of the roots while converting larger molecules into mobile units that plants can absorb [23]. Since nitrogen and phosphorous are both involved in the biosynthesis of secondary metabolites [24–26] AMF may indirectly influence plant defense by increasing their access to these nutrients.

Alternatively, AMF could be directly altering induced defenses by regulating plant gene expression or, possibly, through horizontally transferred genes [27]. Both have been studied in different species, including wheat [28], rice [29], tomato [30], and common beans [31] with relative gene expression of developmental and defensive genes increasing by orders of magnitude in wheat and tomato and horizontal gene transfer often associated with improved resistance against biotic or abiotic stressors [32, 33].

Understanding these mechanisms could be particularly important in agriculture because improved plant defense can reduce the need for pesticides [34, 35], thereby cutting costs and environmental impacts [36]. Cropping systems that promote AMF can be more sustainable and as productive as conventional systems that depend on inputs for the same gains [34]. Additionally, some crop plants with greater AMF colonization have been shown to produce larger amounts of defense compounds, such as in *Nicotiana tabacum* and *Castanospermum austral* [37]. In fact, the defense chemistry of *Plantago lanceolata* differs depending on the species of AMF it interacts with [38] and while AMF (*Rhizophagus irregularis*) significantly influenced polyphenol oxidase production in *Solanum dulcamara*, it did not influence the defense chemistry of *Solanum ptycanthum* [39]. If improved plant nutrition is the primary factor allowing AMF to alter plant defenses, then that service could be filled through means other than promoting AMF (such as fertilization). Alternatively, if soil nutrients and AMF colonization interact to alter chemical defenses, then elucidating this interaction could help farmers to adjust fertilization regimens and agricultural practices to optimize induced defenses in crop plants.

A good model system for investigating these mechanisms is the maize plant (*Zea mays*) and the common maize pest, fall armyworm (*Spodoptera frugiperda*; FAW). In maize the major defense pathways for resisting chewing herbivores involves cascades of genes along the jasmonic acid (JA) and salicylic acid (SA) pathways and the production of downstream defense related compounds that are toxic to insects such as endochitinases that lyse midguts of caterpillars [40], and protease inhibitors that are antinutritive and inhibit caterpillar gut proteases [41]. Studies have shown that feeding by FAW, as well as FAW saliva and frass, elicit changes in expression of genes along both the JA and SA pathways [42–44]. It has also been shown that maize defense compounds are altered by AMF colonization of plant roots [45–47] and by plant fertilization [48–50]. Furthermore, soil legacies left behind by different cover crop species have been shown to differentially alter plant nutrition, AMF colonization, and regulation in JA and SA defense genes in subsequent maize plants, as well as alter the feeding and behavior of FAW larvae toward those maize plants [51].

This study compared the relative contribution of either nutritional inputs or AMF association to defensive responses of maize tissue damaged by FAW. Given that plant response to chewing herbivores involves the expression of genes along both the JA and SA pathways, we used quantitative real time PCR to measure the transcripts of maize defense genes with known associations with FAW performance, *lipoxygenase 3* (*lox3*), *maize protease inhibitor* (*mpi*), and *pathogenesis related protein 5* (*pr5*), after 3 days of feeding. Nutritional digests were also performed to quantify the relative abundance of nitrogen and phosphorous across our treatments. We asked: is the effect of AMF on maize defense simply a result of increased nutrient uptake or do AMF alter defense gene expression by mechanisms unrelated to nutrient uptake?

## Results

**Fig. 1:**
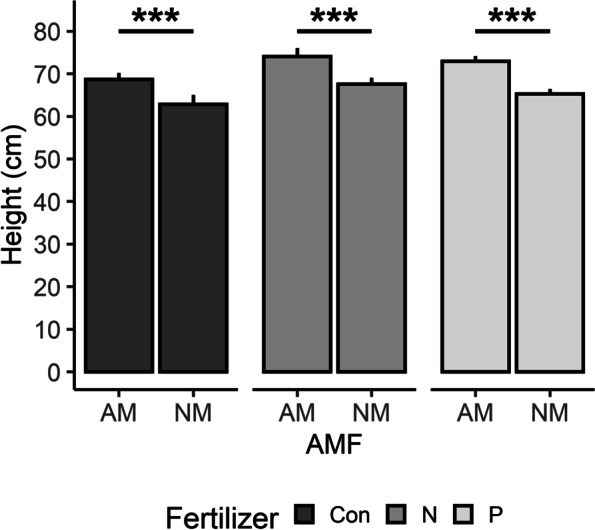
Maize height (cm) when grown with (“AM”) or without (“NM”) arbuscular mycorrhizal fungi (AMF) with standard fertilizer treatment (Con), double the amount of nitrogen (N), or double the amount of phosphorous (P). Maize height was statistically associated with AMF (F = 2738, df = 1, *P* < 0.001). Whiskers represent standard error

Maize plants grown with AMF were taller (*P* < 0.001, Table 1, Fig. 1) and had, on average, fewer leaves than those grown without (*P* < 0.001, Table 1). Fertilizer was also associated with height (*P* = 0.0055) and leaves (*P* = 0.0401) with the nitrogen fertilizer treatment resulting in taller maize and phosphorous with more leaves (Table 1). Neither maize height (*P* = 0.8373) nor leaves (*P* = 0.3545) were associated with the interaction of AMF and fertilizer. Also, dry mass was not statistically altered by AMF (*P* = 0.7528, Table 1) nor the interaction between AMF and fertilizer (*P* = 0.7110), but was altered by fertilizer independently (*P* < 0.001) with control maize having the least dry mass. Furthermore, root staining confirmed AMF were present in 100% of inoculated roots and 0% of the non-inoculated controls.

**Table 1:** Mean ± SE of plant, gene expression, and insect parameters measured by treatment

Parameter	Control	2 × Nitrogen	2 × Phosphorus	AMF	AMF + 2 × Nitrogen	AMF + 2 × Phosphorus
Plant drymass (g)	6.74 ± 1.56	9.83 ± 1.92	9.83 ± 3.25	11.67 ± 3.27	9.30 ± 2.34	11.04 ± 2.88
Plant height (cm)	62.84 ± 2.15	67.57 ± 1.49	65.25 ± 1.13	68.65 ± 1.47	74.05 ± 1.92	72.90 ± 1.20
Number of leaves	7.21 ± 0.27	7.73 ± 0.23	7.77 ± 0.25	7.04 ± 0.19	7.75 ± 0.23	7.14 ± 0.22
Leaf nitrogen (mg/g)	17.79 ± 0.72	17.12 ± 0.46	15.45 ± 0.33	18.46 ± 0.91	18.84 ± 0.70	15.24 ± 0.60
Leaf phosphorus (mg/g)	3.21 ± 0.20	2.52 ± 0.14	3.19 ± 0.12	2.35 ± 0.17	2.52 ± 0.15	2.60 ± 0.24
*lox3* (RQ)	-0.53 ± 0.19	0.19 ± 0.10	-0.73 ± 0.22	-0.34 ± 0.22	-0.56 ± 0.25	-0.68 ± 0.19
*mpi* (RQ)	0.74 ± 0.17	0.46 ± 0.18	0.63 ± 0.10	0.92 ± 0.18	0.91 ± 0.16	0.72 ± 0.21
*pr5* (RQ)	0.08 ± 0.13	0.18 ± 0.11	0.02 ± 0.10	0.49 ± 0.17	0.35 ± 0.18	0.47 ± 0.18
Change in FAW mass (g)	0.08 ± 0.03	0.05 ± 0.02	-0.03 ± 0.03	0.08 ± 0.03	0.04 ± 0.02	0.08 ± 0.02

Nitrogen concentration (mg N/g maize tissue) of the maize leaves had a significant association with fertilizer treatment (*P* < 0.001, Table 1) and was lowest in maize grown in excess P. AMF had no effect on N content (*P* = 0.4060). The opposite was true for phosphorous (mg P/g maize tissue), with AMF increasing total phosphorous (*P* < 0.001, Table 1) but the fertilizer treatments having no effect (*P* = 0.4771). Neither nitrogen (*P* = 0.7099) nor phosphorous (*P* = 0.3225) had a significant interaction effect between AMF and fertilizer treatments. Additionally, higher nitrogen concentration was positively correlated with plant height (*P* < 0.001) and leaves (*P* < 0.001) while phosphorous was not (*P* = 0.1878; and *P* = 0.4754 for height and leaves, respectively).

**Fig. 2:**
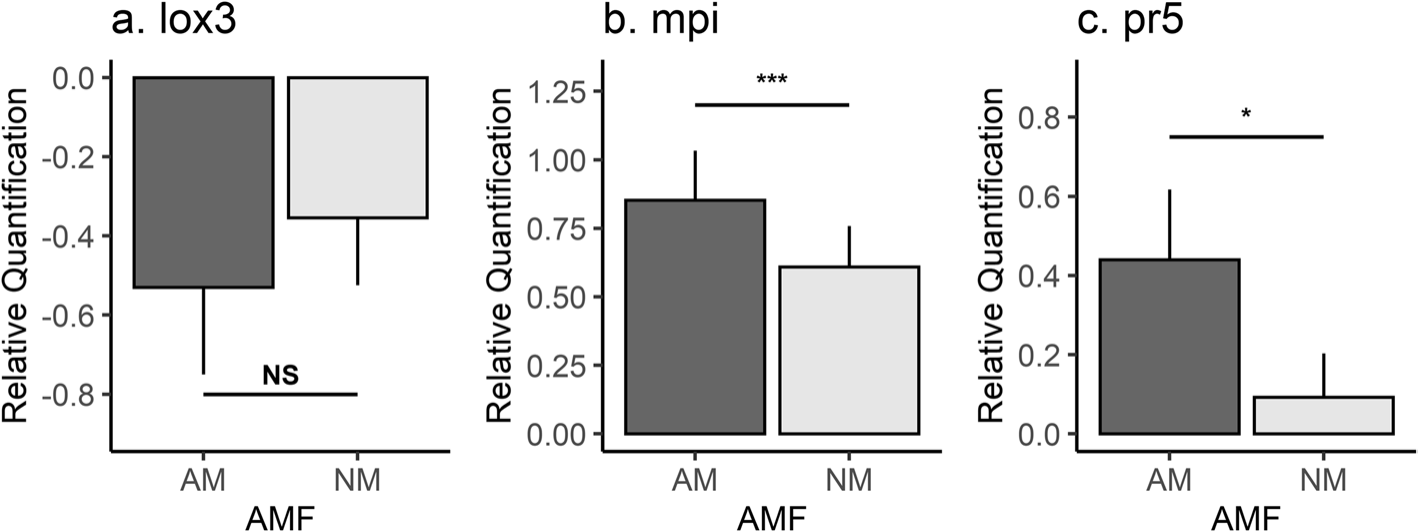
Relative expression and linear relationship to the change in fall armyworm (FAW) mass (g) of **a** *lox3*, **b** *mpi*, and **c** *pr5* genes given the presence (“AM”) or absence (“NM”) of arbuscular mycorrhizal fungi (AMF). Whiskers represent standard error

AMF significantly increased the transcription of *mpi* and *pr5* (Tables 1 and 2; Fig. 2). Of all genes tested, only *mpi* expression was significantly altered by nutrient treatments (*P* = 0.0074, and *P* = 0.0333, for nitrogen and phosphorous, respectively; Table 2) with N and P content both positively associated with its expression. Though *lox3* was not associated with any of the treatments, its expression was, on average, lower in mycorrhizal maize (Table 1, Fig. 2).

**Table 2:** General linear model analyses for AMF, fertilizer, their interaction, foliar N (ppm N), and foliar P (ppm P) on *lox3*, *mpi*, and *pr5* expression in maize responding to FAW feeding. Statistically significant *p*-values are in **bold print**

	*Lox3*			*mpi*			*pr5*		
**Factor**	**df**	**F**	**P**	**df**	**F**	**P**	**df**	**F**	**P**
AMF	1,48	2.301	0.1110	1,48	12.31	** < 0.001**	1,48	3.815	**0.0290**
Fertilizer	2,48	1.377	0.2622	2,48	0.2089	0.8122	2,48	0.1936	0.9003
AMF*Fertilizer	2,48	1.403	0.2557	2,48	0.2466	0.7824	2,48	0.2268	0.7979
ppm N	1,2	1.921	0.3000	1,2	133.43	**0.0074**	1,2	1.9600	0.2965
ppm P	1,2	0.389	0.5967	1,2	28.478	**0.0334**	1,2	1.7537	0.3165

**Fig. 3:**
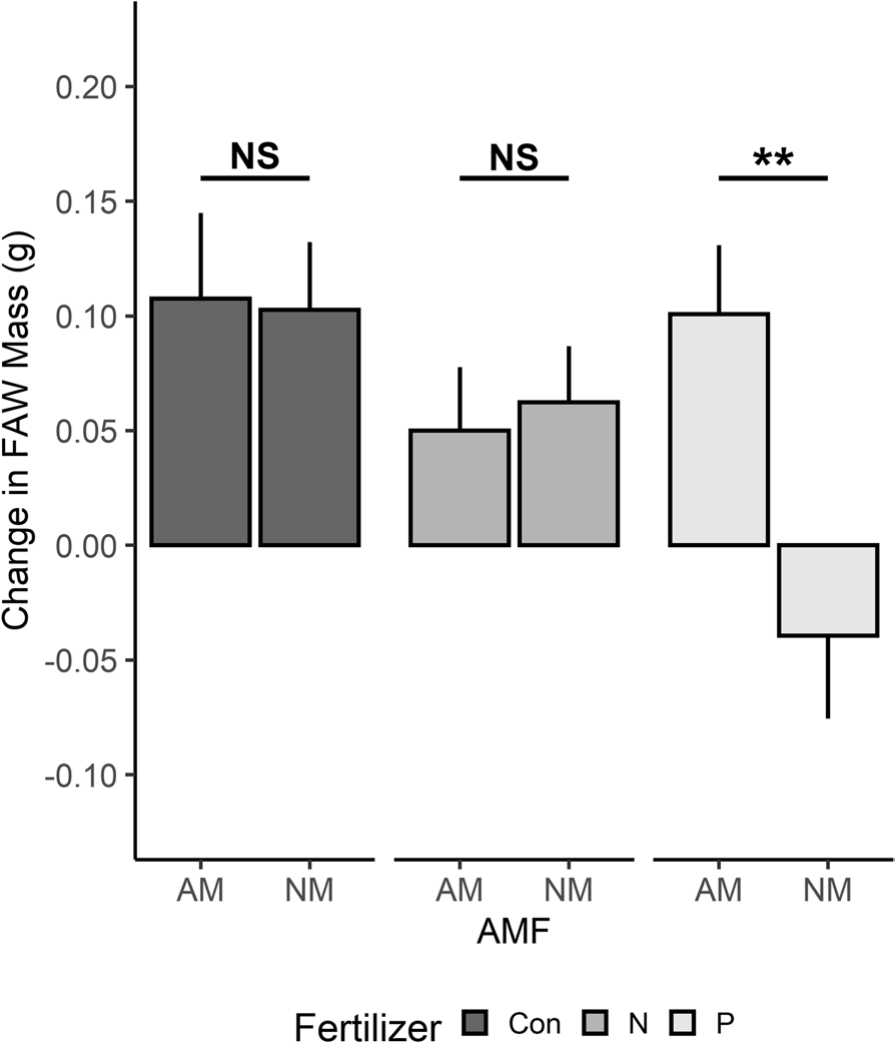
Change in fall armyworm (FAW) mass (g) after fed on maize grown with (“AM”) or without (“NM”) arbuscular mycorrhizal fungi (AMF) with standard fertilizer treatment (Con), double the amount of nitrogen (N), or double the amount of phosphorous (P) for 72 h. Change in mass was statistically associated with AMF treatment (F = 11.46, df = 1, *P* < 0.001) and the interaction of AMF and fertilizer treatments (F = 3.374, df = 2, *P* = 0.0376). Whiskers represent standard error

**Fig. 4:**
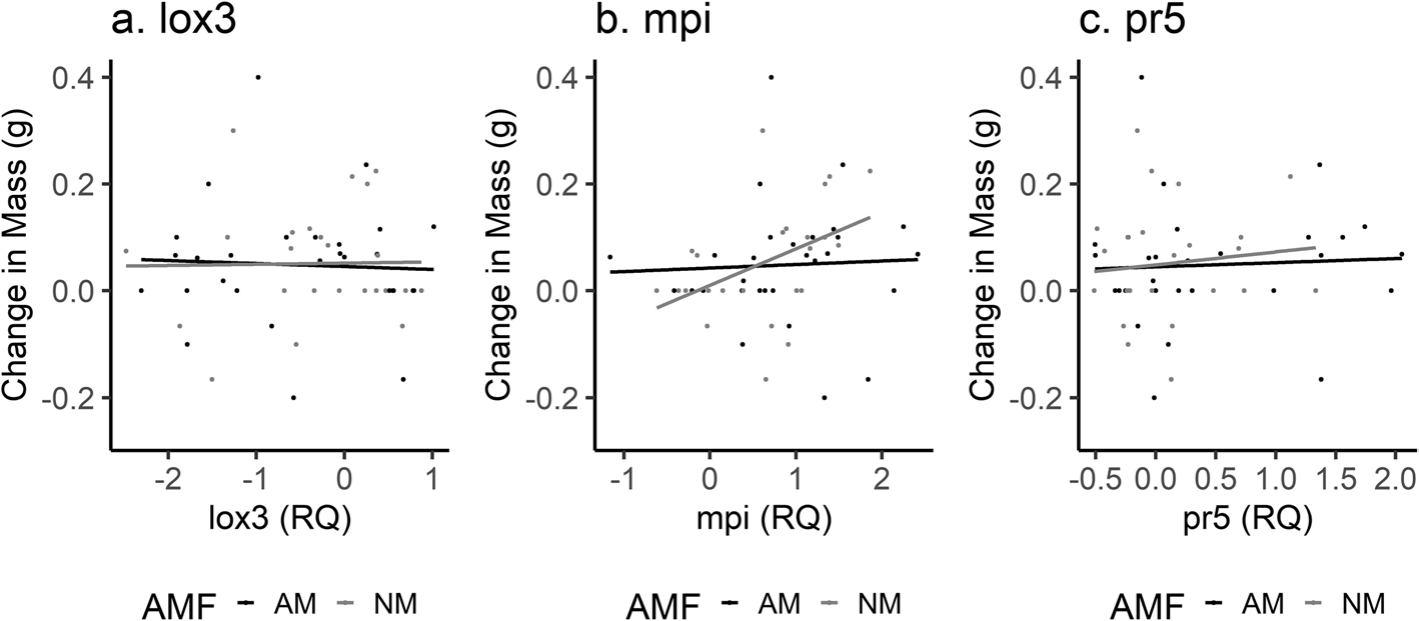
Linear relationship of the relative expression of **a** *lox3*, **b** *mpi*, and **c** *pr5* genes, given the presence (black line) or absence (gray line) of arbuscular mycorrhizal fungi (AMF), to the change in fall armyworm mass (g)

Change in fall armyworm mass was associated with AMF independently (*P* < 0.001, Table 1) and the interaction of AMF and fertilizer (*P* = 0.0376), but had no relationship with fertilizer independently (*P* = 0.0992), specifically, FAW gained more mass on mycorrhizal maize. Furthermore, non-mycorrhizal maize grown in excess P was the only treatment that resulted in a loss in FAW mass (Fig. 3). The only gene associated with FAW mass change was *mpi* (*P* = 0.0114), with FAW gaining more mass on maize with a higher relative quantification of *mpi* (Fig. 4). Neither total leaf nitrogen (F = 0.179) nor phosphorous (*P* = 0.739) concentration (mg/g) were associated with FAW mass change.

## Discussion

This study found that the presence of AMF altered patterns of induced expression of two defense related genes (*mpi* and *pr5*) in maize. In contrast, nitrogen and phosphorous fertilization produced only a slight increase in *mpi*. Our results support the hypothesis that AMF induces the expression of defense related genes in maize through means other than nutritional gains. This could be due to a couple of reasons: 1) AMF primed maize defense prior to FAW herbivory, increasing the number of available transcripts when damage occurred; or, 2) AMF improved the efficiency of the defense response, switching to *mpi* and *pr5* expression quicker. There are many potential routes that AMF could have used to influence the transcription of maize defense genes as nearly anything can influence gene expression. Other work has shown that AMF colonization of plant roots includes the upregulation of specific GRAS transcription factors with at least a portion of the expression including regulatory components that shape root development at arbuscle formations [52] and may allow AMF to influence other aspects of plant physiology.

Increased phosphorus uptake in our AMF-infested plants aligned with previous studies in maize [53]. This increased uptake typically results in more vigorous growth, which was also observed in our study. In contrast, we did not observe an increase in nitrogen uptake in AMF-colonized plants. This result differs from multiple studies showing that AMF colonization can increase N in maize [54–56]. That said, AMF generally contribute to P acquisition by plants more so than N acquisition [57], but the effect can vary based on the relative proportions of N and P in the soil [58]. For example, in excess N, plants may suppress AMF colonization because the relationship would be more parasitic than beneficial in that context [59]. The complex symbiotic and/or antagonistic interactions of the plant-soil ecosphere in a mycorrhizal context could explain why excess nitrogen resulted in the lowest dry mass in our study [60]. Finally, while phosphorous content was highest in mycorrhizal maize in our study, the increase was not the primary influence of defense gene expression.

Though FAW mass was higher in AMF treatments, this does not indicate that the defense response was unsuccessful. The only treatment that resulted in a loss of mass was the non-mycorrhizal maize grown in excess P (Fig. 3), but this was unlikely due to P content since P was higher in mycorrhizal maize. In addition, FAW are known to metabolize plant defenses, excreting altered molecules that switches the response to target pathogens [42, 61, 62]. While the jasmonic and salicylic acid pathways are often both involved in plant defense [63–65], expression of one generally means suppression of the other [66, 67]. The higher expression of *mpi*, which is downstream of JA and is induced by FAW oral secretion, suggests that the appropriate defense molecules were being produced in the AMF treatments [42, 61]. It is also important to note that the caterpillars used in our study were reared on artificial diet for the first few days of their existence [68]. Though significant variation in mass gain was found between fertilizer treatments, it may be more indicative of the amount of diet they had consumed before the assay than the impact of the phytochemical defense, which does not take immediate effect. More specifically, FAW can survive multiple days on digested artificial diet [69]. This diet not only supports continued mass gain but could dilute the potency of defense phytochemicals in the gut. However, while caterpillar survival, and consequently, mass gain, was influenced by cannibalistic behavior during the bioassay [70], plant tissue was consistently damaged in the FAW treatments providing a direct comparison of undamaged versus damaged foliar tissue in the rtPCR analysis.

Our data support previous research that demonstrates AMF not only improve the nutrient acquisition and growth of their host but simultaneously influence their defense response to herbivores. More importantly, augmenting plants with N and P fertilizer did not trigger the same induction of defense related genes as AMF association (Table 1). Though the genes we measured are known to be associated with FAW herbivory, they are a subset of a much larger array of defense potential that AMF may also influence [71]. Deciphering the complex threads that link AMF to the physiology of their hosts could have immense benefits for sustainable agriculture. Given the diversity and ubiquity of mycorrhizal associations, systematic understanding of their influence on plant defense could contribute toward a lower input system that reduces the need for pesticides [72, 73]. The relationships between plants and the organisms that consume them have led to a staggering diversity of phytochemicals with important biological functions [74]. AMF improve the phytochemical toolkit of host plants not only by increasing access to soil compounds but also by affecting the way in which their host responds. Promoting beneficial microbes in agricultural soil is a paradigm shift toward sustainability that we cannot afford to ignore [75].

## Conclusions

Plants biosynthesize targeted defense responses to specific environmental stressors. The current understanding of the biochemical coevolution between maize and FAW has the insect as the front-runner [76, 77]. In our study, the addition of AMF resulted in significant upregulation of defense genes in opposing pathways. Those genes, *mpi*, which targets herbivores, and *pr5*, which targets pathogens [15], were expressed at higher rates in the AMF treatments. Taken together, our results suggest that maize grown with AMF may require less fertilizer and have improved access to both JA and SA defenses. While our work advances the current understanding of how AMF can benefit plant hosts, the minor variation observed within treatments and distinct variation between treatments suggests these relationships are plastic in their impact. Identifying the optimal conditions to draw out the gains provided by AMF to plant hosts could further reduce the need for fertilizer and pesticide inputs in maize production systems.

Ecological intensification of agricultural production will require a complete understanding of the direct and indirect influences AMF have on plant physiology and phytochemical defense. We found that the defense benefit AMF provide to plants does not depend on fertilizer and may allow low-input systems to be protected and highly productive. Since more than 80% of all plant species are obligate mycorrhizal symbionts, there exists massive potential for ecological solutions to the ever-present threats faced in food production.

## Methods and materials

The experiment was conducted in the greenhouse at The Land Institute (Salina, KS) from July 2^nd^, 2019 to August 4^th^, 2019, at 23.9℃ with 16:8 L:D photoperiod. The model species, B73 *Zea mays* (maize) were used for the study. Both rounds consisted of 120 plants with 10 replicates per treatment combination. Treatments consisted of presence/absence of arbuscular mycorrhizal fungi, one of three fertilizer treatments (control, 2 × nitrogen, or 2 × phosphorous), and undamaged vs FAW herbivory in a full factorial design. All plants were hand-watered daily and spot-checked for damage/disease symptoms.

For each of 2 blocks in either round of the experiment, 60 pots of B73 maize seeds were sown with 45 mL of MycoBloom® (MycoBloom LLC, Lawrence, KS) in the AMF treatments or without MycoBloom in the non-AMF treatments. This mixture consists of the AMF species *Claroideoglomus claroideum* (Schenck & Smith)*, Funneliformis mosseae* (Nicolson & Gerdemann)*, Cetraspora pellucida* (Nicolson & Schenck)*, Claroideoglomus lamellosum* (Dalpé, Koske & Tews)*, Acaulospora spinosa* (Walker & Trappe)*, Racocetra fulgida* (Koske & Walker) and *Entrophospora infrequens* (Hall) [78]. Plants were grown in 10.16 × 34.29 cm treepots (Stuewe & Sons Inc, Tangent, OR). Seeds were sown directly into the AMF mix applied on the top of ~ 3,400 cm^3^ of calcined clay (Turface® MVP; Buffalo Grove, IL) then covered with ~ 1 cm of the same medium to protect spores from sunlight. Calcined clay was chosen for this experiment because it allows efficient cleaning and observation of root development and contributes no organic matter or fertilizer to the system [79]. For each fertilizer treatment, a mixture of 24,000 mL of 50% DI water and 50% tap water was made containing 37.2 g Peters Professional 20–20-20 N-P-K general fertilizer (control), 37.2 g Peters Professional + 6.9 g urea (2 × nitrogen), or 37.2 g Peters Professional + 8.82 g Super Phosphorus pellets (2 × phosphorus). Approximately 60 mL of the appropriate solution was added to each treatment replicate twice weekly.

Fall armyworm caterpillars were obtained from Frontier Agriculture Services (Newark, DE). Larvae were approximately 2^nd^—3^rd^ instar (Frontier Agriculture Services, personal communication) when the shipment arrived, and had been previously reared on Frontier General Purpose Lepidoptera Insect Diet (F9772). For each replicate, five larvae were weighed (initial mass) and applied to maize plants at the V5 stage using soft tweezers. A cylindrical aluminum mesh cage was placed around each plant to keep caterpillars on the infested plant. Larvae were left to feed for 72 h then collected, counted, and weighed again (final mass). We then calculated mass change per caterpillar by subtracting final mass from initial mass. At the time caterpillars were collected, approximately 3.5 cm of damaged foliar tissue (undamaged tissue for the non-herbivory controls) were collected from every plant and immediately stored in liquid nitrogen. These frozen plant samples were then stored at -80 °C until RNA extractions were performed.

Roots were trimmed from aboveground tissue and gently shaken to remove Turface. Roots were then rinsed to remove all remaining residue and stored in 70% ethanol in sterile polypropylene specimen containers (Dynarex Corporation, Orangeburg NY). AMF root colonization was confirmed using a root staining protocol adapted from [80]. Fine roots were cut into 1 cm segments and cleared in 10% potassium hydroxide for 3 min, then stained for 3 min with a mixture of vinegar and 5% Sheaffer black ink. Presence or absence of AMF arbuscules was confirmed by scanning 10 random subsections of the cleared/stained roots under a compound microscope at 100X magnification [81].

### Plant defense and nutrient analyses

We measured the relative expression of three plant defensive genes in response to fall armyworm herbivory. Plant are known to primarily induce defense related genes downstream of the jasmonic acid signaling pathway in response to caterpillar feeding [82]. Previous work has shown that transcripts of the gene lipoxygenase 3 (*lox3*) which is involved in the jasmonic acid synthesis pathway is induced post caterpillar feeding in maize [83]. Maize protease inhibitor (*mpi*) which is downstream of jasmonic acid response in maize is also induced by caterpillar herbivory and act as direct defenses against caterpillars [42, 83]. We measured the transcript abundance of both Lox3 and mpi to assess the induction of caterpillar induced defenses in maize with or without mycorrhizal association. [61]. Since it is also well known that salicylic acid response in plants are antagonistic to jasmonic acid responses and are primarily induced by pathogens [84], we also measured the transcript abundance of pathogenesis related protein transcript (*pr5*), which is regulated by salicylic acid [85].

To test gene expression in plants with and without herbivory, total RNA was extracted from the frozen plant tissue samples collected at the end of each round of the greenhouse experiment, following the methods of Ray et al*.*, (2016) [42]. Briefly, 100 mg of leaf tissue was collected from each plant, flash frozen in liquid nitrogen and ground while frozen with metal beads in a GenoGrinder 2000 (OPS Diagnostics). Leaf tissue was collected from damaged leaves in the FAW treatments and undamaged leaves of the same developmental stage in herbivore control plants. RNA was extracted from the ground tissue with 1 mL of TRIzol (Life Technologies) following the manufacturers protocol, quantified with a Nanodrop (Thermo-Fisher Scientific) and 1 ug of total RNA was used to make complementary DNA (cDNA) with a High Capacity cDNA Reverse Transcription kit (Applied Biosystems, USA) and oligo(dt). A Fast Start Universal SYBR Green Master Mix was used to perform qRT-PCR and actin was used as an endogenous reference gene for baseline expression. Four biological replicates were used for each treatment combination. Actin expression in non-mycorrhizal maize grown with the control fertilizer treatment was used as the calibrator control to calculate relative gene expression (RQ) of *lox3*, *mpi*, and *pr5* using the delta-delta Ct method [86]. The primers used to amplify these genes were the same as was used in Ray et. al (2015) [62].

For nutrient analyses, maize tissue samples that were stored at -80 °C were removed from the freezer and dried at 60 °C for 5 days. Due to small volume sample size, dried tissue was ground to < 2 mm, then after undergoing persulfate digestion [87], analyzed colorimetrically for total N [88] and P [89]. Standards were generated using a water matrix with 0–50 ppm for NO3 and 0–10 ppm for PO4, with alfalfa plant standard with known N and P concentration used to confirm accuracy of persulfate digest.

### Statistics

After screening data to ensure they met the assumptions of normality and homogeneity of variance, general linear models with Type II sums of squares were used to test the direct, fixed effects of AMF presence/absence, fertilizer treatments, and the fertilizer by AMF interaction on the following dependent variables: maize height, leaves and dry mass, change in FAW growth, and foliar N and/or P content. Type II SS were used because the data were unbalanced from lost replicates [90]. Relative levels of gene expression were also analyzed in separate linear models as both a dependent and independent variable. General linear model analyses with Type II sums of squares were used to test whether gene expression, as the dependent variable, changed based on AMF or fertilizer treatments and their interaction. The same models were used to test whether foliar N or P content influenced maize height, leaves, dry mass, gene expression, or FAW growth. We also used multiple regression to test whether the change in each gene’s expression (as independent continuous variables) influenced FAW growth. All statistics and figures were done in RStudio [91] using the packages ggplot2 [92] and car [93].

## Data Availability

The datasets used and/or analyzed during the current study are available from the corresponding author on reasonable request.
